# Outbreak of aztreonam–avibactam-resistant NDM-5-producing *Escherichia coli* isolates in a hospital of Paris

**DOI:** 10.1128/spectrum.01672-26

**Published:** 2026-08-13

**Authors:** Véronique Faraut Derouin, Bérénice Souhail, Caroline Charlier, Sébastien Gaultier, Etienne Canoui, Anne Sorieul, Daria Menuet, Hélène Poupet, Laurence Armand-Lefèvre, Eve-Marie Walle, Delphine Desbois, Alexandra Doloy, Philippe C. Morand, Claire Poyart, Romain Coriat, Asmaa Tazi, Hedi Mammeri

**Affiliations:** 1 Hôpitaux Universitaires Paris Centre, Site Cochin, Service d’Hygiène hospitalière, Assistance Publique Hôpitaux de Paris 378848 , Paris, France; 2 Hôpitaux Universitaires Paris Centre, Site Cochin, Equipe mobile d’infectiologie, Assistance Publique Hôpitaux de Paris 378848 , Paris, France; 3 Université Paris Cité 555089, Paris, France; 4 Centre national de référence français et Centre collaborateur de l’OMS pour la Listeria, Institut Pasteur 27058, Paris, France; 5 FHU PREM’Impact, Paris, France; 6 Hôpitaux Universitaires Paris Centre, Site Cochin, Service de Bactériologie, Assistance Publique Hôpitaux de Paris 378848 , Paris, France; 7 Hôpitaux universitaires Paris Nord Val de Seine, Site Bichat, Laboratoire de Bactériologie, Assistance Publique Hôpitaux de Paris 378774 , Paris, France; 8 INSERM UMR 1137 IAME, Université Paris Cité 555089, Paris, France; 9 Center hospitalier de Rambouillet, Laboratoire d’analyses de biologie médicale 707216 , Rambouillet, France; 10 Université Paris Cité, INSERM U1016, CNRS UMR8104, Institut Cochin 55648, Paris, France; 11 Department of Gastroenterology and Digestive Oncology, Cochin Hospital, Assistance Publique - Hôpitaux de Paris 26930, Paris, France; 12 Centre National de Référence des Streptocoques, Hôpital Cochin 26935, Paris, France; JMI Laboratories, North Liberty, Iowa, USA

**Keywords:** duodenoscope, ERCP, ST405, outbreak, avibactam, aztreonam, resistance, cefiderocol, carbapenem, PBP3, DHA, NDM-5, *Escherichia coli*

## Abstract

The emergence of aztreonam–avibactam resistance among NDM-producing *Escherichia coli* represents a major therapeutic and infection control concern. Four cases of aztreonam–avibactam-resistant (AZAR) NDM-producing *E. coli*, recovered from three blood cultures and one rectal surveillance culture in a hospital of Paris, prompted an investigation based on whole-genome sequencing. The three isolates responsible for bloodstream infection, which were clonally related, belonged to the ST405 and produced NDM-5, DHA-1, CTX-M-15, and TEM-1 β-lactamases. They also had a penicillin-binding protein 3 (PBP3) modified by the insertion of four amino acids (YRIK). On the other hand, the isolate recovered from the rectal swab belonged to another sequence type (ST2083). It produced NDM-5, CMY-42, and TEM-1 β-lactamases and had an altered PBP3 by the insertion of four other residues (YRIN). Epidemiological investigation identified endoscopic retrograde cholangiopancreatography as a potential risk factor, leading to the detection of three additional patients infected with the ST405 clone after exposure to the same duodenoscope. The outcome of the bloodstream infections was favorable except for one patient. In total, seven patients infected or colonized by AZAR NDM-5-producing *E. coli* were identified over a 5-month period in 2025, six with duodenoscope exposure.

**IMPORTANCE:**

This study underlines the emergence of aztreonam–avibactam resistance among NDM-producing *Escherichia coli* isolates in France. To our knowledge, this report describes the first clonal outbreak due to aztreonam–avibactam-resistant NDM-producing *E. coli*.

## INTRODUCTION

Carbapenems are considered last-resort antibiotics for treating patients with severe infections caused by multi-drug-resistant Gram-negative bacteria. The emergence of carbapenem resistance is a major public health concern, and the World Health Organization has identified carbapenem-resistant Enterobacterales as critical-priority bacteria (1).

Carbapenem resistance may arise due to the acquisition of plasmid-borne carbapenemases, including NDM-type enzymes that are one of the most common, particularly in *Escherichia coli* (2). *E. coli* producing NDM-type metallo-β-lactamases (MBL) is resistant to all clinically available β-lactams but aztreonam (ATM)–avibactam (AVI) and cefiderocol (FDC), even in combination with extended-spectrum β-lactamases (ESBLs) or AmpC-type enzymes. From a therapeutic perspective, the Infectious Diseases Society of America (3) and, more recently in 2024, the European Society of Clinical Microbiology and Infectious Diseases (4) have recommended these antibiotics as reference treatments for infections caused by MBL-producing Enterobacterales. The primary target of ATM and FDC is penicillin-binding protein 3 (PBP3), an essential transpeptidase responsible for *E. coli* cell division. Insertion mutations in PBP3 lead to reduced susceptibility to ATM-AVI and FDC, a crucial step toward pan-β-lactam resistance (5, 6). While amino acid insertions in PBP3 alone mediated slightly reduced susceptibility to ATM-AVI, their combination with AmpC-type β-lactamases, such as CMY-type enzymes, can confer high-level resistance to ATM–AVI (6–8).

Over a 6-week period in 2025, four NDM-producing *E. coli* isolates resistant to ATM–AVI and FDC were recovered in a Parisian hospital (Paris, France). Molecular characterization revealed that three of these isolates were clonally related. An epidemiological investigation conducted by the hospital’s infection control team identified a duodenoscope as the most likely source of cross-contamination. A review of all patients who had undergone endoscopic retrograde cholangiopancreatography (ERCP) with this device, between the last microbiological safety assessment and its withdrawal from service, identified three additional infected patients. To the best of our knowledge, this is the first reported hospital-acquired clonal outbreak caused by an ATM–AVI-resistant (AZAR) NDM-producing *E. coli* strain. In this study, we provide the detailed genomic analysis of this outbreak.

## MATERIALS AND METHODS

### Bacterial identification and epidemiological investigation

The study started with the isolation of four NDM-producing *E. coli* strains (AZAR1, AZAR2, AZAR3, and AZAR4) at hospital setting 1 located in the Paris region, in France. Matrix-assisted laser desorption ionization-TOF (Bruker Daltonics, Germany) was carried out for bacterial identification at the species level. NDM-type carbapenemase production was confirmed using the NG-Carba5 immunochromatographic assay (NG-Biotech, Guipry, France). These NDM-producing isolates were notable for their resistance to the association ATM-AVI (MICs 8 mg/L). This unusual phenotype prompted an investigation combining whole-genome sequencing (WGS) with an epidemiological investigation. Multiple sources and transmission routes were considered. A medical record review revealed that a history of ERCP procedures involving the use of a duodenoscope was common among AZAR2, AZAR3, and AZAR4 cases. All patients who underwent an ERCP procedure with this device were notified of their potential exposure to carbapenem-resistant *E. coli*. Once suspected, the duodenoscope was sequestered and cultured. Duodenoscope reprocessing procedures were directly observed, including in-room manual precleaning and high-level disinfection with an automated endoscope reprocessor.

### Antimicrobial susceptibility testing

Minimum inhibitory concentrations (MICs) were determined using gradient test strips according to the manufacturer’s instructions (bioMérieux, Marcy-l’Étoile, France, and Liofilchem, Montpellier, France, for cefepime-zidebactam) on Mueller–Hinton agar plates (Sanofi Diagnostics Pasteur, Paris, France). The MICs of piperacillin-tazobactam and FDC were determined using the broth microdilution method (Biocentric, Bandol, France). Results were interpreted according to the European Committee on Antimicrobial Susceptibility Testing definition of clinical breakpoints (9).

### WGS and comparative genomic analysis

We used short-read WGS to describe the molecular characteristics of the initial ATM-AVI-resistant *E. coli* isolates as well as those recovered after the epidemiological investigation. The MasterPure Gram-Positive DNA (Fischer Scientific, Illkirch, France) was used to extract and purify genomic DNA following the manufacturer’s instructions. Qubit and the HS (high sensitivity) dsDNA assay kit (Thermo Fisher Scientific) were used to quantify genomic DNA for each extract, following the manufacturer’s instructions. The complete genome sequences of the isolates were obtained by Illumina sequencing with a NextSeq 500 sequencer at the Mutualized Platform for Microbiology (Institut Pasteur, Paris, France). SPAdes software (version 3.10.1) was used for read assembly (10). Quality metrics of assemblies were assessed using QUAST v5.0.2. The depth of coverage and the biometric parameters (N50 and L50) of the final assemblies are presented in Table S1. The genome was annotated using the MicroScope platform (http://www.genoscope.cns.fr/agc/microscope) (11). The ResFinder web server (https://genepi.food.dtu.dk/resfinder) was used to identify antimicrobial resistance-encoding genes. A phylogenetic analysis was performed on assembled genomes of clinical isolates together with the genome of the ST405 *E. coli* LYO25 (accession number DABGKQ010000000) as a reference, using CSI Phylogeny 1.4 server (Call SNPs & Infer Phylogeny; https:cge.cbs.dtu.dk/services/CSIPhylogeny) with default settings, to construct an SNP distance matrix (12).

### Typing methods

The genome sequence-inferred serotype and the sequence type (ST) were identified with software available from the Center for Genomic Epidemiology (SerotypeFinder 2.0, with a threshold of 85% identity and a minimum length of 60%, and MLST 2.0, respectively; www.genomicepidemiology.org/) by uploading the genome of *E. coli* AZAR1 and *E. coli* AZAR2. Virulence genes were identified with software from the Center for Genomic Epidemiology (VirulenceFinder 2.0, with a threshold of 90% identity and a minimum length of 60%). The phylogenetic group was determined using the *in silico* Clermont phylotyper (http://clermontyping.iame-research.center/) (13).

Nucleotide sequence accession numbers for whole-genome shotgun projects of *E. coli* AZAR1, AZAR2, AZAR3, AZAR4, AZAR5, AZAR6, and AZAR7 have been deposited at GenBank under accession numbers JBRYRV000000000, JBRYSD000000000, JBRXXJ000000000, JBRYRZ000000000, JBRYSC000000000, JBRYSA000000000, and JBRYSB000000000, respectively.

## RESULTS

### Epidemiological investigations

Over a 6-week period in 2025, four patients (AZAR1, AZAR2, AZAR3, and AZAR4) were identified, including three clinical isolates and one rectal surveillance isolate (Table 1). These strains exhibited an unusual resistance to both ATM–AVI and FDC (Table 2). This unexpected finding prompted an investigation to better understand and prevent the transmission of these isolates.

**TABLE 1:** Clinical features of the patients infected or colonized by aztreonam-avibactam resistant NDM-producing *E. coli* isolates

Patient’s name	Age (year old)	Underlying diseases	Endoscopic retrograde cholangiopancreatography performed at the hospital setting 1	Sequence type of the *E. coli* isolate	Nature of the positive sample	Colonization versus infection	Antibiotic treatment of the ATM–AVI-resistant NDM-producing *E. coli* infection	Outcome of the *E. coli* infections (j30)
AZAR1	47	Cholangiocarcinoma	None	ST2083	Rectal swab (Hospital setting 1)	Colonization	None	Not applicable
AZAR2	58	Cholangiocarcinoma	Yes	ST405	Blood + bile (Hospital setting 1)	Tumoral cholangitis complicated with non-drained hepatic abscesses (<3 cm)	Day 1: colistin (9 MUI/24 h) + fosfomycine (12 g/24 h) Day + 2: tigecycline (200 mg/24 h) + fosfomycine (12 g/24 h) Day + 14: eravacycline (1 mg/kg/24 h) + fosfomycine (12 g/24 h) (total 4 weeks of treatment)	Favorable
AZAR3	50	Metastatic colorectal cancer	Yes	ST405	First episode: Blood (Hospital setting 1)	Tumoral cholangitis	Tigecycline (200 mg/24 h) + fosfomycin (12 g/24 h) Total: 7 days of treatment	Relapse
					Second episode: Blood (Hospital setting 1)	Postoperative intra-abdominal abscess, radiological drainage	Colistin (9 MUI/24 h) + fosfomycin (12 g/24 h) Total: 12 days of treatment	Favorable
AZAR4	58	Pancreatic ductal adenocarcinoma	Yes	ST405	Rectal swab (Hospital setting 1)	Colonization	None	Not applicable
AZAR5	67	Pancreatic ductal adenocarcinoma	Yes	ST405	First episode: Blood (Hospital setting 2)	Tumoral cholangitis	Day 1: ceftazidime – avibactam + aztreonam Day 4: tigecycline (200 mg/24 h)	Relapse
					Second episode: Blood (Hospital setting 2)	Bloodstream infection and pylephlebitis	Day 1: Eravacycline (50 mg/12 h) + Colistine (9 MUI/24 h) Day 7: Eravacycline (90 mg/12 h) + Colistine (9 MUI/24 h) + Fosfomycine (12 g/24 h) Total: 4 weeks of treatment	Fatal
AZAR6	64	Pancreatic ductal adenocarcinoma	Yes	ST405	Blood (Hospital setting 3)	Tumoral cholangitis	Co-trimoxazole	Favorable
AZAR7	52	Pancreatic ductal adenocarcinoma	Yes	ST405	Bile (Hospital setting 1)	Colonization	None	Not applicable

**TABLE 2:** MIC values, β-lactamase content, and PBP3 modification of the *E. coli* strains

		MIC (mg/L)		
		AZAR2, AZAR3, AZAR4, AZAR5, and AZAR6	AZAR1	
β-Lactamase content		NDM-5, DHA-1, CTX-M-15, and TEM-1	NDM-5, CMY-42, and TEM-1	
PBP3 modification		YRIK	YRIN	
Amoxicillin		>256	>256	
Amoxicillin-clavulanate^*a*^		>256	>256	
Piperacillin-tazobactam^*b*^		>256	>256	
Ceftazidime		>256	>256	
Cefotaxime		256	256	
Cefepime		128	256	
Cefepime-zidebactam^*c*^		0.5	0.5	
Cefiderocol		8	8	
Ertapenem		>256	>256	
Imipenem		32	32	
Meropenem		64	64	
Aztreonam-avibactam^*d*^		8	8	
Gentamicin		>256	>256	
Amikacin		>256	2	
Colistin		0.5	0.5	
Tigecycline		0.125	0.25	
Eravacycline		0.125	0.125	
Co-trimoxazole		>256	>256	
Levofloxacin		>256	>256	
Ciprofloxacin		>256	>256	
Fosfomycin		1	2	

^*a*^ Clavulanate at 2 µg/mL.

^*b*^ Tazobactam 4 µg/mL.

^*c*^ Zidebactam was fixed at a 1:1 ratio.

^*d*^ Avibactam at 4 µg/mL.

**Fig 1:**
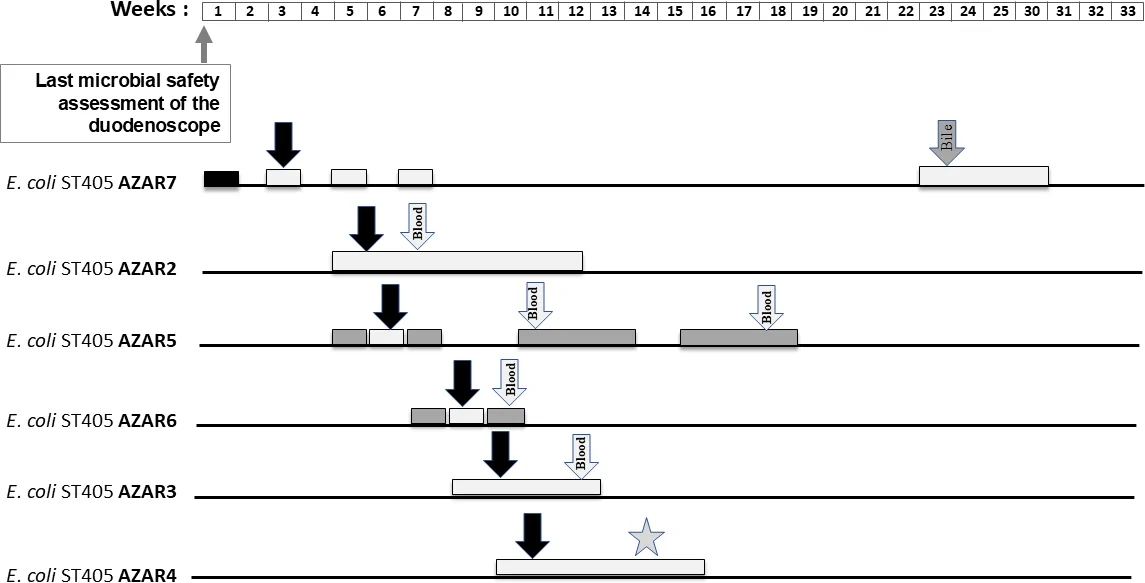
Timeline of the outbreak for the six patients infected by the aztreonam–avibactam-resistant *E. coli* ST405 clone. The time scale at the top of the figure represents the weeks between the last microbiological safety assessment of the duodenoscope and the occurrence of bloodstream infections, bile duct infection, or rectal colonization. The black box represents hospitalization in a foreign country (Mauritius); white boxes correspond to the hospitalizations in the Parisian hospital setting 1; gray boxes correspond to the hospitalizations in the Parisian hospital setting 2 or 3; black arrows represent ERCP using the suspected duodenoscope in the hospital setting 1; white arrows represent bloodstream infections; gray arrow indicates bile duct infection; gray star corresponds to positive rectal swab.

Epidemiological tracing revealed no temporal overlap of the patient rooms or wards. On the other hand, three patients, AZAR2, AZAR3, and AZAR4, who were treated at hospital setting 1, had an ERCP at that facility and were exposed to the same duodenoscope. The hospital’s operational hygiene team listed all the patients who underwent ERCP with the same device, between the last microbiological safety assessment and the time of its withdrawal from service. Among the patients who were discharged home after ERCP, none responded to the notification letter sent by the hygiene team, and no rectal screening could be performed. Only patient AZAR7 was readmitted to the hospital for cholangitis, and the ST405 clone was isolated from its bile sample. Among the patients who underwent ERCP, two were immediately transferred to hospitals 2 and 3 (AZAR5 and AZAR6, respectively), and an AZAR strain was recovered from their blood culture before the epidemiological investigation (Fig. 1). The clinical features of the patients are summarized in Table 1.

All patients suffering from bloodstream infection were first treated with ATM-AVI upon identification of the NDM-producing *E. coli* strains. Tigecycline, fosfomycin, colistin, or eravacycline were used once antimicrobial susceptibility testing revealed resistance to all available β-lactams (Table 2). The outcome of the bloodstream infections was favorable for all patients except one (AZAR5), whose death was attributable to carbapenem-resistant *E. coli* infection (Table 2).

Duodenoscope reprocessing procedures were directly observed, and no deviations from national and manufacturer-provided guidelines were identified. Similarly, the organization of all regulatory monitoring was in place. The duodenoscope was promptly removed from service upon suspicion of contamination. Its microbiological assessment, using non-selective culture media, was performed by the hospital’s infection control team and failed to detect *E. coli*. The duodenoscope was subsequently sent to the biomedical engineering department for disassembly and cleaning, and it was permanently withdrawn from service.

### Susceptibility testing

Table 2 presents the MIC values of the AZAR *E. coli* isolates. Strains were resistant to all β-lactams, including cefiderocol and aztreonam-avibactam isolates, but were susceptible to the novel combination cefepime-zidebactam, which is not yet available in France. The AZAR2, AZAR3, AZAR4, AZAR5, AZAR6, and AZAR7 isolates shared the same pattern of resistance and were resistant to all aminoglycosides, whereas the AZAR1 strain remained susceptible to amikacin. All strains were susceptible to colistin, tigecycline, eravacycline, and fosfomycin.

### Genotyping analysis and resistance determinants

According to the genome-inferred typing, the AZAR1 strain belonged to phylogroup B1, serotype O188:H6, and sequence type ST2083, whereas the AZAR2, AZAR3, AZAR4, AZAR5, AZAR6, and AZAR7 isolates belonged to phylogroup D, serotype O102:H6, and sequence type ST405. *In silico* screening of resistance genes showed that AZAR1 carried *bla*_NDM-5_, *bla*_CMY-42_, *bla*_TEM-1_, *aph(6)-Id*, *aph(3”)-Ib*, *aac(3)-IId*, *aadA5*, *aac(6’)-ib-cr*, *mph(A*), and *sul2* genes, whereas AZAR2 to AZAR7 carried *bla*_NDM-5_, *bla*_CTX-M-15_, *bla*_DHA-1_, *bla*_TEM-1_, *rmtB*, *mph(A*), *qnrB4*, *sul1*, *tet(B),* and *dfrA17* genes. The seven clinical isolates produced the metallo-β-lactamase NDM-5 and a plasmid-mediated cephalosporinase (CMY-42 for AZAR1 and DHA-1 for all the other strains; Table 2). AZAR2 to AZAR7 also produced the CTX-M-15 ESBL and the TEM-1 penicillinase, whereas AZAR1 only co-produced TEM-1 (Table 2). AZAR1 was resistant to all aminoglycosides except amikacin due to phospho- or acetyl-transferases such as APH(6)-Id, APH(3”)-Ib, AAC(3)-IId, AAC(6’)-Ib-cr, or to adenylyl-transferases such as AADA5 (14, 15), whereas AZAR2 to AZAR7 were resistant to all aminoglycosides due to the production of the 16S rRNA methyltransferase RmtB (16). AAC(6’)-Ib-cr and QnrB4 contributed to quinolone resistance in the AZAR1 strain and the AZAR2 to AZAR7 isolates, respectively (17). The *sul1* and *dfrA17* genes were responsible for cotrimoxazole resistance in the AZAR2 to AZAR7 isolates (15). In addition, the genes for macrolide resistance *mph(A*) were also identified in these latter strains (18). Point mutations associated with quinolone resistance were detected in the chromosome-borne *gyrA* (S83L and D87N) and *parC* (S80I) genes for all isolates (19).

Moreover, strains exhibited an in-frame duplication of a 12-nucleotide stretch in *ftsI*, resulting in the addition of YRIN in the PBP3 for AZAR1, and YRIK in the PBP3 for AZAR2, AZAR3, AZAR4, AZAR5, AZAR6, and AZAR7, respectively (Table 2).

### Clonal relationship

To examine the genetic relatedness of isolates, we performed core genome SNP analyses, which revealed the clonal dissemination of the AZAR2, AZAR3, AZAR4, AZAR5, AZAR6, and AZAR7 strains. Despite the absence of a clear consensus on the maximum cut-off to define clonality, the SNP distance between the ST405 that were isolated during our outbreak was very low (maximum 18 SNP distance). On the other hand, the SNP distance between our isolates and the reference ST405 *E. coli* LYO25, which was recovered in 2016 in France and was not epidemiologically related, was very high (>2,100 SNPs; Table S2).

### Virulence factors

Comparison with the virulence factor database (https://cge.cbs.dtu.dk/services/VirulenceFinder/) showed that our ST405 clone harbored at least 19 virulence genes. These determinants mainly clustered into three functional categories: iron acquisition systems (*irp2*, *fyuA*, *chuA*, *iucA*, *iucC*, *iutA*, and *sitA*) (20), adhesins and fimbrial structures (*csgA*, *fimH*, *papA*, *papC*, and *yeh*) (21, 22), and toxins or factors involved in epithelial interaction (*hlyE*, *yghJ*, *fdeC*, and *eilA*) (21, 23–25). In addition, genes involved in immune evasion and capsule export (*tra T*, KpsE) were detected, further supporting the extraintestinal pathogenic potential of this clone (21, 26).

## DISCUSSION

In total, seven patients infected or colonized by AZAR NDM-5-producing *E. coli* were identified over a 5-month period in 2025. Six had been exposed to a duodenoscope within a short 6-week period. No additional AZAR NDM-5-producing *E. coli* isolates were recovered in the hospital setting 1 after the removal of the duodenoscope. All duodenoscope-related outbreak strains were clonally related and belonged to ST405.

Resistance to ATM-AVI among sporadic NDM-producing *E. coli* ST405 isolates, due to amino acid insertion in PBP3 and coproduction of AmpC β-lactamase, was previously reported in Sweden, France, and India (27–29). To the best of our knowledge, our report describes the first outbreak due to a clonal dissemination of an NDM-producing *E. coli* strain that was resistant to all available β-lactams, including ATM-AVI and FDC. Apparent transmission was associated with exposure to a duodenoscope, which bacterial assessment using non-specific cultural media failed to detect *E. coli*. It was not retrospectively possible to grow carbapenem-resistant *E. coli* from the duodenoscope using enrichment media containing carbapenem nor to attempt an amplification of the *bla*_NDM-5_ gene by PCR, since it was rapidly sent to the biomedical engineering department for disassembly and cleaning maintenance. Therefore, no microbiological evidence could support our hypothesis, which constitutes the limitation of the study. However, the patients AZAR2, AZAR3, AZAR4, AZAR5, AZAR6, and AZAR7 had been exposed to the same duodenoscope, confirming the pattern of the exposure. Moreover, no additional carriers were identified among patients not exposed to the duodenoscope in our hospital. It remains, therefore, a credible assumption. It is likely that the patient AZAR7 was the initial source of the ERCP-related outbreak at our facility (Fig. 1). Five patients were identified as actively infected with the outbreak-related ST405 strain; among them, four developed bloodstream infections, and one asymptomatic carrier was identified. No deviations from manufacturer-provided guidelines in duodenoscope reprocessing procedures were found during the field investigation.

Other institutions that have experienced similar outbreaks have reported that a single duodenoscope can transmit drug-resistant organisms to patients over an extended period despite adherence to recommended disinfection procedures. Humphries et al. reported a duodenoscope-related outbreak of *bla*_OXA-232_-expressing *Klebsiella pneumoniae* isolates in the Los Angeles County Department of Public Health in the US (30). Epstein et al. described an outbreak of 39 case patients after exposure to duodenoscopes contaminated with NDM-producing *E. coli*. In both studies, no reprocessing breaches or duodenoscope defects were identified. The complicated design of duodenoscopes makes cleaning difficult. It appears that these devices have the potential to remain contaminated with pathogenic bacteria even after recommended reprocessing is performed (31).

Occurrence of NDM-5-producing *E. coli* has increased worldwide (32, 33). ST405, which is one of the predominant sequence types among NDM-5-producing *E. coli* isolates, together with ST167, ST410, and ST361, is considered a high-risk clone based on its rapid spread and its association with virulence and multidrug resistance (34–38). Indeed, ST405 *E. coli* clones were reported to possess a broad spectrum of virulence factors and are considered as extraintestinal pathogenic strains (phylogroup D) (39). This virulence pattern may explain why bloodstream infections occurred among 71% of our patients, often several weeks after ERCP, consistent with the ability of this clone to colonize and invade the epithelial cells lining the bile duct.

Moreover, *E. coli* isolates belonging to ST405 have been implicated in the dissemination of genes encoding ESBLs, primarily CTX-M enzymes, carbapenemases (NDM), and 16S methylases such as ArmA and RmtB (40–42). In our study, the ST405 clone produced NDM-5 that differs from NDM-1 by two amino acids (Val-88-Leu and Met-154-Leu), responsible for increased resistance to extended-spectrum cephalosporins and carbapenems (43). However, NDM-5 alone, even overproduced (44), cannot confer ATM–AVI resistance. Target modifications resulting from a four-amino acid insertion in PBP3 represent a key mechanism contributing to both FDC and ATM-AVI resistance. Molecular modeling has shown that the introduction of four residues (YRIK/YRIN/YRIP) at position 333 induces conformational alterations that hinder the binding of β-lactam agents with bulky side chains, such as aztreonam and ceftazidime, at the binding site on the PBP3 protein (6). Long et al. showed that among 159,341 publicly available high-quality *E. coli* genome assemblies, 2.01% contain PBP3 insertions, predominantly a tetrapeptide duplication of amino acids 334–337 (8). However, modification in PBP3 was not able to confer resistance alone to CFD and ATM–AVI. Indeed, Alm et al. found that amino acid insertions in PBP3 alone mediated slightly reduced susceptibility to ATM-AVI (6). However, by cloning and plasmid-curing experiments, they showed that the combination of insertions in PBP3 combined with the plasmid-mediated β-cephalosporinase CMY-42 conferred high-level resistance to ATM–AVI (6). An experimental study showed that the presence of CMY-42, which derived from CMY-2 by a single amino acid substitution at position 211 on the Omega loop, but not CMY-2, reduced the susceptibility to avibactam in combination with aztreonam or ceftazidime (5). Sadek et al. showed that all NDM-5-producing *E. coli* isolates categorized as “resistant” to ATM–AVI carried plasmid-borne *bla*_CMY_ β-lactamase genes, with *bla*_CMY-42_ allele being the predominant allele (45). In our study, all isolates but one carried the *bla*_DHA-1_ gene, thus suggesting that DHA-1 could also promote FDC and ATM-AVI resistance when combined with PBP3 alteration. Additional experiments are warranted to ascertain this hypothesis.

### Conclusion

The increasing incidence of carbapenem-resistant *E. coli* ST405 is of major concern given its capacity for rapid dissemination and outbreak potential. Hospital settings constitute a particularly conducive environment for the spread of ST405, owing to intense antibiotic selective pressure and the concentration of highly vulnerable patients. The combined effect of PBP3 alteration and DHA-1 production might overcome the protective activity of AVI toward ATM, leading to clinically relevant resistance in *E. coli*. To our knowledge, our report describes the first clonal outbreak due to AZAR NDM-producing *E. coli*. Moreover, it shows that most of the ATM–AVI-resistant NDM-5 *E. coli* ST405 were isolated during bloodstream infections in our hospital setting, thus underscoring their clinical relevance. Moreover, our findings suggest the potential for carbapenem-resistant *E. coli* transmission following ERCP and the critical importance of stringent infection measures and duodenoscope surveillance.

## Data Availability

The whole-genome sequencing data used and analyzed during this study are publicly available with the GenBank access numbers indicated in the Materials and Methods section.

## SUPPLEMENTAL MATERIAL

Tables S1 and S2 spectrum.01672-26-s0001.docx Table S1: The biometric parameters of the final assemblies of NGS. Table S2: The SNPs matrix between the genomes of AZAR strains.

ASM does not own the copyrights to Supplemental Material that may be linked to, or accessed through, an article. The authors have granted ASM a non-exclusive, world-wide license to publish the Supplemental Material files. Please contact the corresponding author directly for reuse.
