# Supplementary material for: Outbreak of aztreonam–avibactam-resistant NDM-5-producing Escherichia coli isolates in a hospital of Paris
Source: Microbiol Spectr. 2026 Aug 13;14(9):e01672-26. doi: 10.1128/spectrum.01672-26 (PMC13532323; doi:10.1128/spectrum.01672-26)
Supplement: Supplementary file 1 [file spectrum.01672-26-s0001.docx]

**Supplemental Files**

**Table S1.** Deep of coverage and biometric parameters of the final assemblies

| Isolates | Depth of coverage | N50 ^1^ | L50 ^2^ |
| --- | --- | --- | --- |
| AZAR3 | 100 | 90,412 | 16 |
| AZAR1 | 125 | 170,896 | 10 |
| AZAR4 | 87 | 90,412 | 15 |
| AZAR6 | 252 | 105,767 | 15 |
| AZAR7 | 229 | 118,170 | 15 |
| AZAR5 | 297 | 97,531 | 16 |
| AZAR2 | 97 | 94,397 | 16 |

^1^ the *N50* is defined as the sequence length of the shortest contig at 50% of the total assembly length.

^2^ the *L50* is defined as count of smallest number of contigs whose length sum makes up half of genome size.

**Table S2.** SNPs matrix between the genomes of the AZAR isolates included in this study

| AZAR isolates | AZAR1 | AZAR2 | AZAR3 | AZAR4 | AZAR5 | AZAR6 | AZAR7 |
| --- | --- | --- | --- | --- | --- | --- | --- |
| AZAR1 | 0 | 41,770 | 41,771 | 41,765 | 41,766 | 41,763 | 41,763 |
| AZAR2 | 41,770 | 0 | 11 | 5 | 10 | 13 | 13 |
| AZAR3 | 41,771 | 11 | 0 | 8 | 15 | 16 | 18 |
| AZAR4 | 41,765 | 5 | 8 | 0 | 7 | 8 | 10 |
| AZAR5 | 41,766 | 10 | 15 | 7 | 0 | 11 | 11 |
| AZAR6 | 41,763 | 13 | 16 | 8 | 11 | 0 | 12 |
| AZAR7 | 41,763 | 13 | 18 | 10 | 11 | 12 | 0 |
